# Integrative diagnosis, biological observations, and histopathology of the fig cyst nematode *Heteroderafici* Kirjanova (1954) associated with *Ficuscarica* L. in southern Italy

**DOI:** 10.3897/zookeys.824.26820

**Published:** 2019-02-12

**Authors:** Elena Fanelli, Alessio Vovlas, Simona Santoro, Alberto Troccoli, Giuseppe Lucarelli, Nicola Trisciuzzi, Francesca De Luca

**Affiliations:** 1 Istituto per la Protezione Sostenibile delle Piante (IPSP), Consiglio Nazionale delle Ricerche (CNR), S.S. Bari, Via G. Amendola 122/D, 70126 Bari, Italy Consiglio Nazionale delle Ricerche Bari Italy; 2 A. P. S. Polyxena, Via Donizetti 12, 70014 Conversano, Bari, Italy Unaffiliated Bari Italy; 3 Hortoservice, Via S. Pietro 2, Noicattaro, Bari, Italy Hortoservice Bari Italy; 4 Centro Ricerca Sperimentazione e Formazione in Agricoltura (CRSFA), Locorotonto, Bari, Italy Centro Ricerca Sperimentazione e Formazione in Agricoltura Bari Italy

**Keywords:** cyst-forming nematode, embryogenesis, histopathology, identification, phylogeny, SEM morphology

## Abstract

Morpho-biological notes and histopathology, based on LM and SEM observations, of the fig cyst nematode *Heteroderafici* isolated from *Ficuscarica* roots, collected in home and public gardens of Apulia region, southern Italy, are described and illustrated. Seventy-five localities throughout the Apulia region were sampled and one-quarter of the sampled localities had fig roots infested with *H.fici*, with population densities ranging from 44 to 180 cysts/100 ml of soil. All attempts to detect *H.fici* on ornamental *Ficus* spp. as well as on imported bonsai in Italy were unsuccessful. Morphometric characters of the Italian population conform to those of the type and re-description populations reported for *H.fici*. Molecular analysis using ITS, D2–D3 expansion domains of the 28S rRNA, and the partial 18S rRNA sequences of *H.fici* newly obtained in this study matched well with the corresponding sequences of *H.fici* present in the GenBank database. Phylogenetic trees confirmed and supported the grouping of *H.fici* in the Humuli group. *Heteroderafici* completes its embryogenic development in 14–16 days at 25 °C. Post-invasion development and maturity in the roots of *F.carica* seedlings is completed in 64–68 days at 25–28 °C with juveniles and adults showing different parasitic habits, being endoparasitic and semi-endoparasitic respectively. The establishment of permanent feeding sites that consist of the formation of large syncytia causes anatomical modification of vascular elements and general disorder in the root stelar structures. Syncytia structures associated with mature females showed different degrees of vacuolisation, numbers of syncytial cells, and contained nuclei and nucleoli which were constantly hypertrophied.

## Introduction

The fig cyst nematode *Heteroderafici* Kirjanova, 1954 was described from roots of rubber plants, *Ficuselastica* Roxb. ex Hornem from China by Kirjanova (1954) and 35 years later a re-description, based on observations of females, males, cysts, and juveniles collected in USA and Pakistan on *Ficuscarica* Linneaus, 1753 was given by Golden et al. (1988). Di Vito and Inserra (1982) demonstrated the pathogenicity of *H.fici* from *F.carica* seedlings in pots. However, the impact of the nematode on adult fig trees has never been ascertained. Therefore, *H.fici* is not considered a major pest and its narrow host range is limited to *Ficus* species only. Subbotin et al. (2010) summarised the known occurrence of the fig cyst nematode from Germany, Greece, Hungary, Italy, the Netherlands, Norway, Poland, Portugal, Russia, Spain, Yugoslavia, China, Georgia, Iran, Turkey, Uzbekistan, Australia, New Zealand, United States (California, Florida, Louisiana, Maryland, and Virginia), Brazil, Algeria, and South Africa. More recently, *H.fici* was also found for the first time on *F.carica* maintained in a nursery in Canada (Sun et al. 2017). *Heteroderafici* together with *H.vallicola* Eroshenko, Subbotin & Kazachenko, 2001 and *H.mediterranea* Vovlas, Inserra & Stone, 1981 are reported as parasites of woody plants. In particular, *H.fici* is a worldwide parasite of ornamental and cultivated *Ficus* species (*F.carica*, *F.elastica*, *F.rubiginosa* Desf. ex Vent., *F.benghalensis* Linneaus, 1753, *F.lyrata* Warb., *F.australis* Wild., and *F.benjamina* Linneaus, 1767) (Baldwin and Mundo-Ocampo 1991, Evans and Rowe 1998; Wohlfarter et al. 2011). Based on classical morphology and host data, *H.fici* has been variously placed with members of the Schachtii group (Mulvey and Golden 1983, Baldwin and Mundo-Ocampo 1991) or in the Avenae group (Stone 1975). Subbotin et al. (2001) and recently De Luca et al. (2013), placed *H.fici* within the Humuli group characterised by bifenestral vulva in most species of the group, few or absent bullae, very weak underbridge, and long vulval slit situated in a cleft between the thickened vulval lips.

Preliminary investigations indicated that *H.fici* was rather widespread in southern Italy and in particular in the Apulia region. The main objectives of the present research were to: (i) obtain additional information on distribution, morphology, and molecular details of *H.fici* from *F.carica*, (ii) establish the phylogenetic relationships among *Heterodera* species closely related to *H.fici*; (iii) obtain additional biological information (embryogenic and post-invasion development); and (iv) to provide morpho-biological details on the host-parasite relationships of this nematode species in fig-nematode-feeding sites and describe the *F.carica* host responses to the nematode parasitism.

## Material and methods

### Nematode population

In the framework of the project “Urban Phytonematology” and on the basis of occasional records of *H.fici* on edible figs (*Ficuscarica*), an extensive survey, including more than 75 root and soil samples, was conducted in commercial orchards in several localities of the Apulia region and in private and public gardens of Bari city, southern Italy, in late May 2016. Twenty-five bonsai *Ficus* spp. from several import/export nurseries working with Asiatic *Ficus* plants, still in their original pots, were included in the present survey. The nematode population selected and used for the present study was collected at Bari University Campus (40°06'72"N, 16°52'54"E).

### Nematode diagnosis

For diagnosis and identification, cysts and eggs were collected directly from infested roots, whereas second-stage juveniles, mature cysts, and males were extracted from soil by the flotation method (Coolen 1979), and cysts by the routine sieving-decanting method (Fenwick 1940). Cysts recovered from infected root tissues were mounted in glycerine. For morphological and diagnostic studies, specimens were glycerine-infiltrated and preserved by conventional methods (Seinhorst 1966), and cysts were fixed and mounted in lactophenol. Glycerine-infiltrated specimens were used for studies on morphometric traits and illustrations with camera lucida and an AmScope microscope. All measurements are given in micrometres unless otherwise stated.

Terminal cone structures of mature cysts were prepared for scanning electron microscopy (SEM) observations. Specimens fixed in formaldehyde (4% solution) were dehydrated in an ethanol gradient, critical-point dried, sputter-coated with gold, and observed according to procedures described by Abolafia et al. (2002).

### Molecular characterization

Individual cysts were crushed with a sterile micro-spatula under a stereo-microscope and the second stage juveniles (J2) were collected. Genomic DNA was extracted from fifteen J2s as described by De Luca et al. (2013). The crude DNA isolated from each individual nematode was directly amplified. The ITS1-5.8S-ITS2 regions were amplified using the forward primer TW81 (5’-GTTTCCGTAGGTGAACCTGC-3’) and the reverse primer AB28 (5’-ATATGCTTAAGTTCAGCGGGT -3’) (Joyce et al. 1994); the D2A-D3B expansion segments of 28S rRNA gene was amplified using the primers D2A (5’-ACAAGTACCGTGGGGAAAGTTG-3’) and the D3B (5’-TCGGAAGGAACCAGCTACTA-3’) (Nunn 1992); the 18S rDNA was amplified using the 18SnF (5’-TGGATAACTGTGGTAATTCTAGAGC-3’) and 18SnR (5’-TTACGACTTTTGCCCGGTTC-3’) (Kanzaki and Futai 2002). PCR cycling conditions used for amplification were: an initial denaturation at 94 °C for 5 min, followed by 35 cycles of denaturation at 94 °C for 50s, annealing at 55 °C for 50s and extension at 72 °C for 1 min and a final step at 72 °C for 7 min. The size of the amplification products was determined by comparison with the molecular weight marker ladder 100 (Fermentas, St. Leon-Rot, Germany) following electrophoresis of 10 μl on a 1% agarose gel.

PCR products of the ITS containing region, the 18S rRNA gene and the D2-D3 expansion domains from three individual nematodes were purified using the protocol given by the manufacturer (High Pure PCR elution kit, Roche, Germany). Purified DNA fragments were cloned and sequenced, in both directions, at MWG-Eurofin in Germany.

### RFLP analysis

Ten μl of the ITS containing amplicons of *H.fici* from southern Italy were digested with the following restriction enzymes: *Alu* I (Roche), *Hae* III (Roche), *Pst* I (Roche) and *Rsa* I (Roche) (5 U of enzyme for each digestion) at 37 °C overnight. The digested DNA fragments were loaded onto 2.5% agarose gel and visualized by gel red staining gel. All gel images were stored digitally.

### Phylogenetic analysis

A BLAST (Basic Local Alignment Search Tool) search at NCBI (National Center for Biotechnology Information) was performed in order to confirm nematode origins and species (Altschul et al. 1997). The newly obtained sequences for ITS containing region, the D2-D3 expansion domains of 28S rRNA gene and 18S rRNA gene were aligned using MAFFTv.7 software (Katoh and Standley 2013) with default parameters with the corresponding published gene sequences of *Heterodera* species. Sequence alignments were manually edited using BioEdit in order to improve the multi-alignment. Outgroup taxa for each dataset were chosen according to the results of previously published data (De Luca et al. 2013). Phylogenetic trees, obtained for ITS dataset, the D2-D3 expansion domains and 18S rRNA gene were performed with the Maximum Likelihood (ML) method using MEGA version 6 software (Tamura et al. 2013). ML analysis under a general time reversible and a gamma-shaped distribution (GTR + G) model for ITS, 28S and 18 S datasets was carried out. Phylogenetic trees were bootstrapped 1000 times to assess the degree of support for the phylogenetic branching indicated by the optimal tree for each method. The newly obtained sequences were submitted to GenBank with the following accession numbers: LT996913 for the ITS region; LT996915 for the D2–D3 expansion domains; LT996914 for the 18S rRNA gene.

### Embryogenic development

The embryogenic development of *H.fici* was studied in Petri dishes, using single-celled eggs obtained (deposited) from newly formed cysts, washed in distilled water, placed in 2% water agar, and maintained in an incubator at 26 ± 2 °C. Microscopic observations and micrographs were taken at six-hour intervals during the first week and daily for the second week.

### Post-invasion development

The duration of post-invasion development was determined on fig (*F.carica*) seedlings transplanted in pots containing 250 ml of pasteurised sand, and inoculated, five days later, by using 1250 juveniles per pot. Inoculated young plants were maintained in a glasshouse at 26–28 °C. Invasion and nematode development was studied by stereoscope observations in acid fuchsin stained roots at seven-day intervals (Fig. 4). The maximum developmental stage observed was that of young cysts containing more than 25 eggs.

### Host-suitability test

Host studies were made in a glasshouse at 26–28 °C using common Mediterranean fruit trees as possible hosts (almond, apple, orange, edible and wild fig, apricot, grapevine, loquat, walnut, and pistachio) and transplanted on naturally infested soil with estimated initial population 1500 juveniles + eggs per pot and exposed to the nematode for a three-month period. Plants were removed from pots, the roots washed free of adhering soil, and the nematode populations were recorded.

### Histopathology

The histological changes induced by *H.fici* were studied in nematode-infected fig roots. Infected and healthy root segments were fixed for 48 hours in formalin-acetic acid-ethanol (FAA) solution, dehydrated in tertiary butyl alcohol, and embedded in 56–58 °C melting-point paraffin. Embedded tissues were sectioned transversely and longitudinally in 10–12 µm thick with a rotary microtome, stained with safranin and fast-green, and mounted in dammar xylene for microscopic examinations (Johansen 1940).

## Results

### Heterodera fici

Taxon classificationAnimaliaTylenchidaHeteroderidae

Kirjanova, 1954

**Figure 1.**
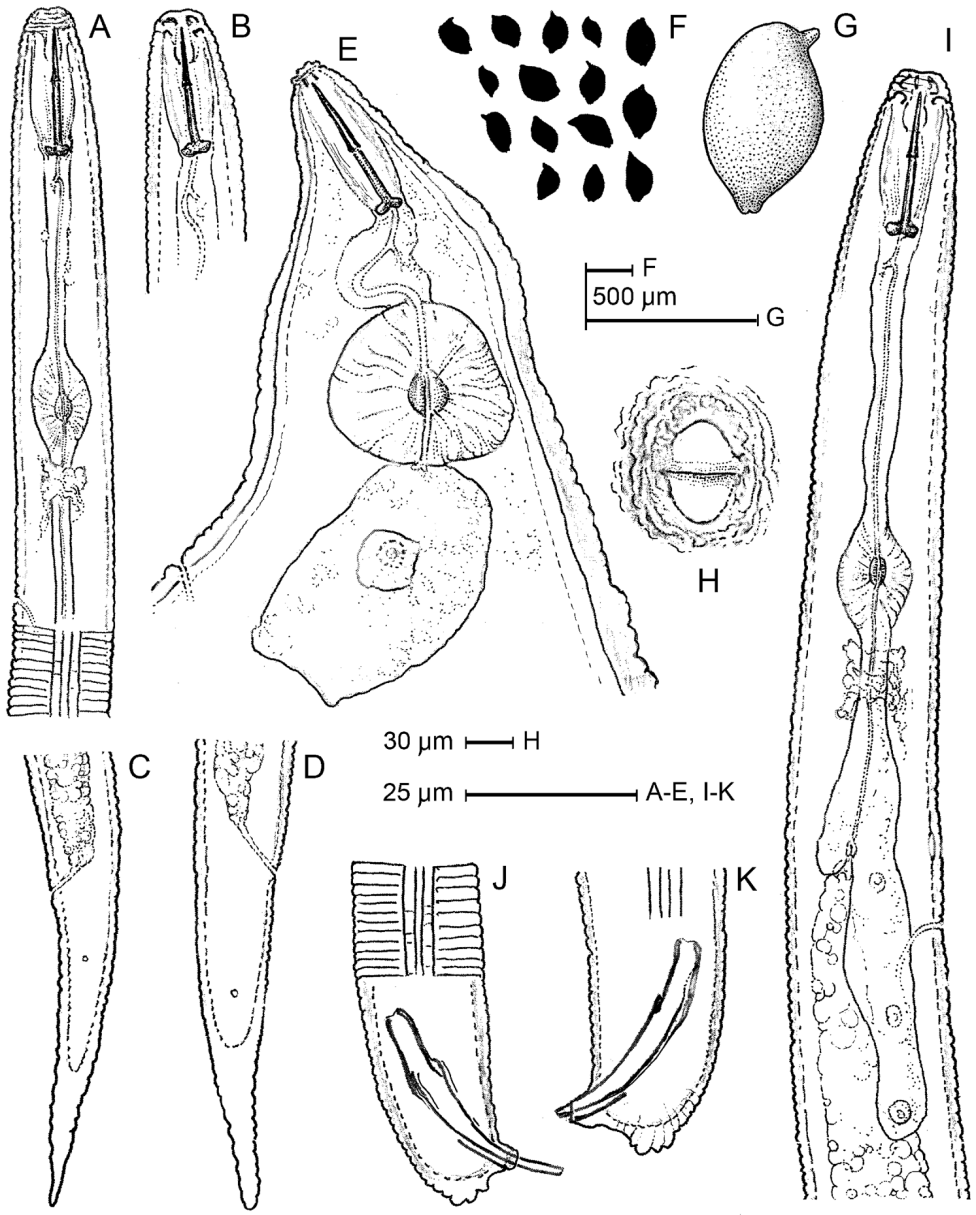
Line drawings of *Heteroderafici* from Italy. **A, B** Anterior body portions of second stage juvenile **C, D** Second stage juvenile tail **E** Female anterior region **F, G** Cyst shape **H** Fenestral structures **J, K** Male tail, showing spicules and cloacal tube **I** Male pharyngeal region.

**Figure 2.**
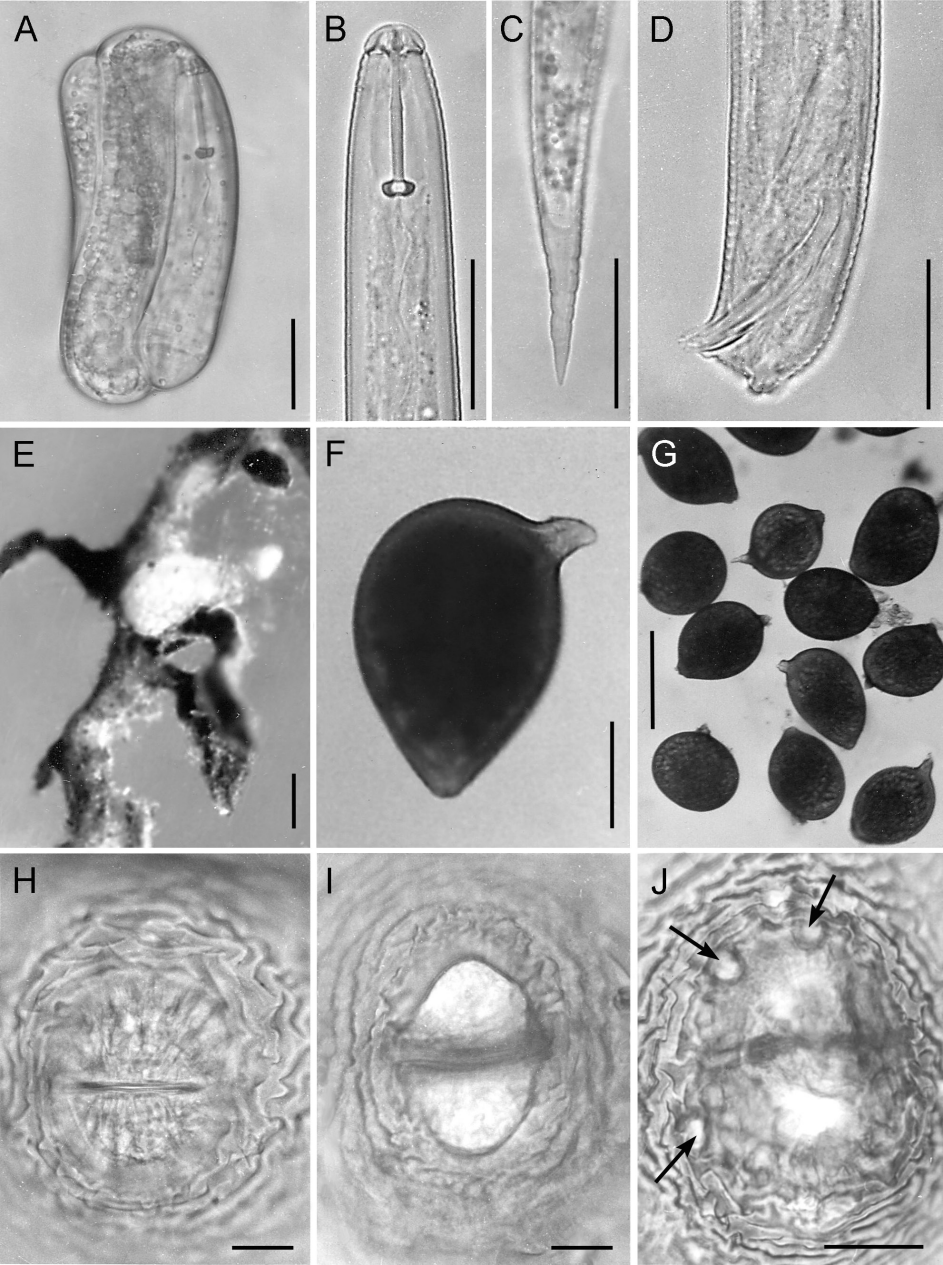
LM micrographs of *Heteroderafici* from Italy. **A** Embryonated egg with evident second stage juvenile stylet **B** Second stage juvenile anterior end **C** Second stage juvenile tail **D** Male tail with the characteristic tail tip **E** Females on *Ficuscarica* roots **F** whole body of newly formed cyst **G** Females and cysts **H-J** Vulval cone structures, with clear illustration of vulval slit (in **H**), fenestral area (in **I**) and bullae (in **J**). Scale bars: 20 µm (**A-D, H-J**); 200 µm (**F**); 500 µm (**E, G**).

**Figure 3.**
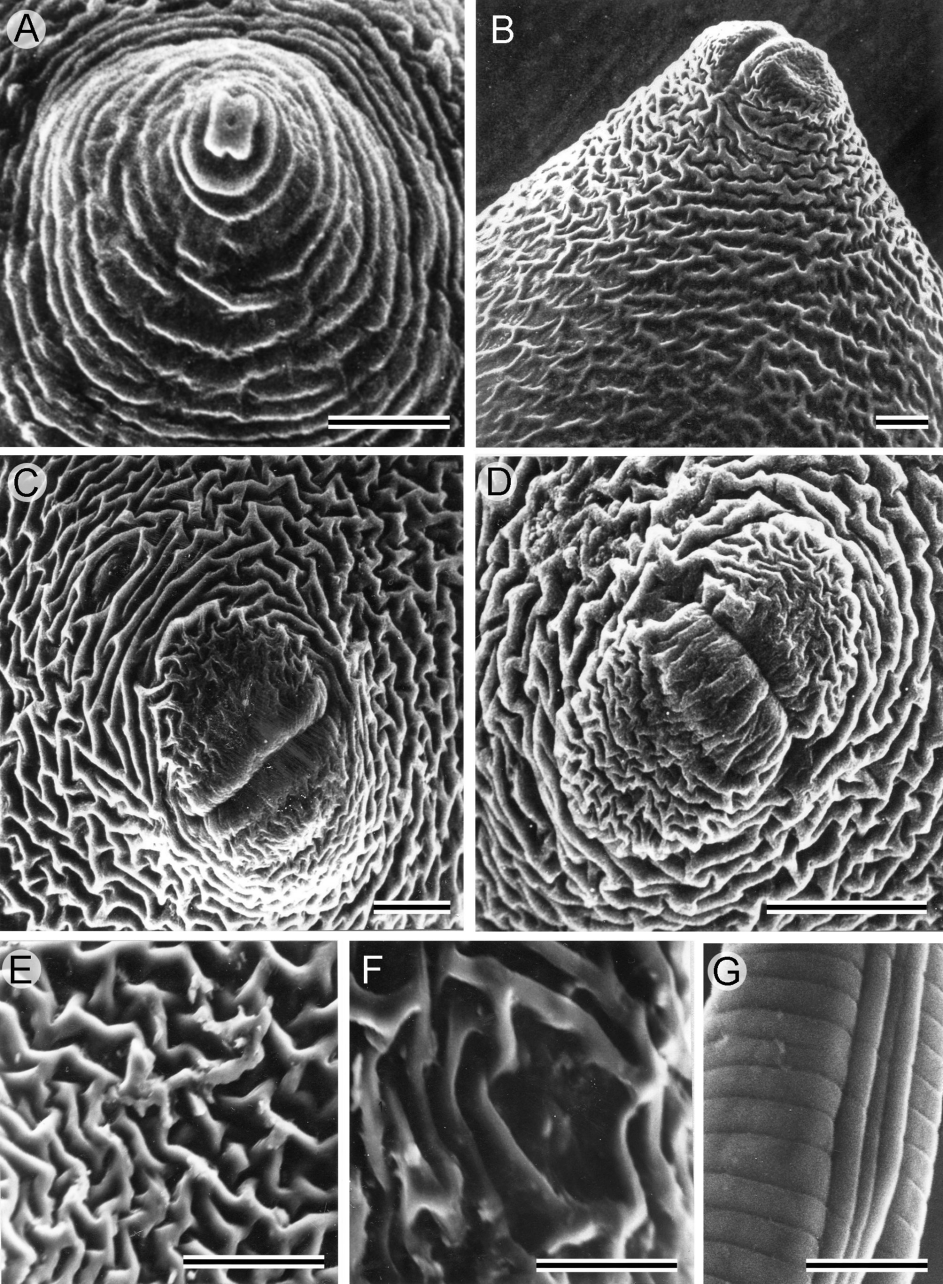
SEM photographs of the main cyst diagnostic characters of *Heteroderafici* from Italy. **A** Female anterior end **B** Lateral view of terminal cone **C, D** Fenestral area and anus **E** Maze-like cyst cuticular ornamentation **F** Cyst anal area **G** Male lateral fields. Scale bars: 10 µm (**A, G**); 20 µm (**B–F**).

**Figure 4.**
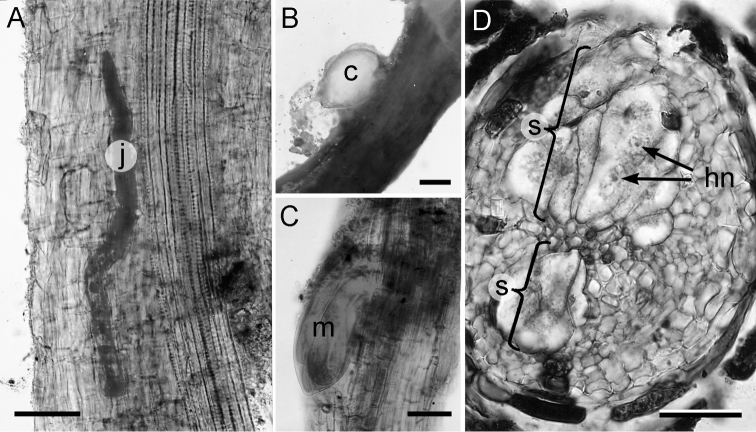
Post invasion development of *Heteroderafici* on *Ficuscarica* roots. **A** Second stage juvenile within cortical layer, oriented in parallel position to the root axis **B** Newly formed cyst with gelatinous egg sac **C** Male inside 4^th^ stage cuticle **D** Different sized syncytia (S) induced by female (the larger one), and by male. Abbreviations: j = juvenile; m = male; c = cyst; hn = hypertrophied nuclei. Scale bars: 50 µm (**A, C**); 200 µm (**B**); 100 µm (**D**).

Figures 1 , 2 , 3 , 4

#### Nematode population.

The fig cyst nematode *H.fici* was recovered in our survey in established commercial fig orchards (more than 30 years old) as well as in private and public gardens. High infection rates were observed ranging from 44 to 180 cycts/100 ml of soil, 12–36 cysts per g of roots; 1.2–1.6 eggs – J2 /ml of soil, thus suggesting that the nematode might be causing damage. Furthermore, *H.fici* was detected in one-quarter of the 75 localities sampled throughout the Apulia region. All sampling attempts to detect *H.fici* from ornamental *Ficus* spp. as well as from imported bonsai in Italy were unsuccessful.

#### Description.

***Measurements.*** See Table 1. *Nematode diagnosis.* Detailed morphometric observations of the Italian population based on second-stage juveniles, male body length and characteristic of tail, stylet length, adult female and cyst shape, and vulval cone features (Figs 1–4, Table 1) agree very well with most of the original morphometric data and the redescription.

**Table 1.** Morphometrics of an Italian population of *Heteroderafici* isolated from roots and soil around roots; specimens mounted in water agar temporary slides (Measurements are in µm).

Character*	Females	Cysts	Males	juveniles
n	15	16	12	20
Body length (excl. neck) (L)	355–638	420–680	750–880	330–416
Max. body width (W)	230–422	280–550	25–26	17–20
L/W	1.2–1.5	1.1–1.5		
Neck length	75–126	–		
Fenestral length	–	46–72		
Fenestral width	–	28–46		
Vulva slit	–	35–44		
Bullae	–	Present, small		
Stylet length	23–28	–	27–31	20–22
Dorsal gland orifice (DGO)	4–5	–	5–6	4–5
Anterior end to centre of median bulb	60–65	–	80–106	64–76
Excretory pore from anterior end	132–138	–		
Head to end of pharyngeal gland lobe			200–275	136–156
Tail length			7.5–8.2	39–52
Hyaline tail portion			–	18–24
No of lines in lateral fields			4	4
Spicule length			26–32	–
Gubernaculum			7–8	–
a			30–38	17–22
b			2.5–4.5	2.4–3.2
c			102–128	8–9

* All characters and indexes (a, b and c) are specified in Siddiqi (2000).

Observing the morphology (Figs 1–4), as well the metric data of the Italian population (Table 1), together with molecular comparison, we conclude that our *H.fici* belongs to the Humuli group and is distinguished from similar species by a combination of morphological and morphometric characteristics; it differs from all other members of the Humuli group (*H.humuli* Filipjev, 1934, *H.ripae* Subbotin, Sturhan, Rumpenhorst & Moens, 2003, *H.vallicola* Eroshenko, Subbotin & Kazachenko, 2001, and *H.litoralis* Wouts & Sturhan, 1996 by ambifenestrate rather than bifenestrate cysts and a longer vulval slit (43–48 vs. <40 μm), and by the prominent nipples on the male tail tip, which are regularly annulated and obtusely rounded in all other members of the group.

***Females.*** Body basically lemon-shaped. Neck elongate, protruding vulval cone prominent. Cyst cuticle with zigzag pattern. Vulval cone well developed. Egg sac present, but few eggs deposited. Cuticular striae, extending to vulval slit are present at fenestral area (Fig. 2H).

***Cysts.*** Body light to dark brown, basically lemon-shaped, neck and vulval cone distinct. Neck protruding, curved laterally. Cuticle thin, without sub-crystalline layer. External wall pattern at mid-body with interlocking ridges, forming zigzag pattern. Terminus of vulval cone with strongly developed zigzag ridges surrounding vulval slit and fenestra. Fenestra ambi-fenestrate, vulval slit equal in length to bridge. Few but distinct bullae are present. Anus distinct, on a depressed area surrounded by continuous cuticle edge/margin (Figs 2F–J, 3F).

***Males.*** Body slender, vermiform, slight ventral curvature. Cuticular annulation prominent. Lateral field areolated, with four incisures. Labial region slightly offset, hemispherical, with three or four annuli. Labial framework heavily sclerotised. Tail short, obtusely rounded, four prominent nipples on tail tip. Spicules arcuate, tapering distally. Gubernaculum slightly curved ventrally (Figs 2D, 3G).

***J2* (*Second stagejuveniles*).** Body vermiform; tapering at both extremities, more marked posteriorly. Cuticular annulation prominent. Lateral field with four incisures. Labial region slightly offset, rounded, with three or four annuli. Labial framework moderately sclerotised. Stylet well developed, basal knobs rounded, directed slightly anteriorly. Median bulb ovoid. Pharyngeal lobe usually distinct, with three large nuclei and overlapping anterior part of intestine. Tail long, tapering, terminus rounded. Anus distinct. Phasmid openings small but distinct, 11–14 μm, posterior to anus and anterior to middle of tail. Hyaline tail region half of tail length (Fig. 2B, C).

***Embryogenesis.*** Mean dimensions of single-celled and embryonated eggs were 40 × 98 µm (Fig. 2A). The first cleavage was equatorial and the two-blastomeres stage appeared after 14–18 hours. The second and third divisions were also transverse and the four-cell stage phase was obtained after three days. Forty-eight hours later, rapid cell division resulted in the formation of multicell eggs. The gastrula stage was observed during days 9–12. First and second-stage juveniles appeared in 11–13 and 14–16 days, respectively, and were coiled three or four times within the eggshell. The embryogenic development was basically the same as in *H.schachtii* Schmidt, 1871 and *H.mediterranea* (Mulvey and Golden 1983, Vovlas and Inserra 1983).

***Post-invasion development.*** Post root-invasion development of *H.fici* on *Ficuscarica* roots was completed in about 64–68 days at 26–28 °C. Invasion and nematode development stages were recorded by stereoscope observations on acid fuchsin-stained roots at seven-day intervals (Fig. 4). The maximum developmental phase observed was that of young cysts containing more than 25 eggs. The first period (24–26 days after inoculation) was utilized by the parasitic specimens for exploration, penetration, and selection of the feeding site (Fig. 4A). The second stage juveniles stop penetration and start to feed on the initial feeding cells by using a sedentary endoparasitic feeding position. Observations at 50–56 days after inoculation revealed that the 3^rd^ moult was completed and that sexual differentiation occurred. The swollen posterior portion of sexually mature nematodes (piriform females and 4^th^ stage vermiform males still inside the cuticle) protrudes from the root. Fourth-stage females and males were observed one week later (Fig. 4C). Lemon-shaped mature females were observed eight weeks after invasion. The post infection period was concluded about 8 weeks later. No new cysts were observed.

***Histopathology.****Heteroderafici* establishes permanent, fully-developed feeding sites on *F.carica* roots and reaches maturity in 64–68 days. Histological examination of sectioned healthy and nematode-infected (Fig. 4) fig roots showed that infection by *H.fici* can cause cellular alterations in the cortex, endodermis, pericycle, and vascular parenchyma tissues of fig roots. Observations of cross-sections of *H.fici* infected roots indicated that the nematode can induce the formation of both cortical and endodermal syncytia. In many cases, the nematode female only penetrated one to three layers of the cortical root cells without reaching the stele. In these roots, the nematode established a permanent feeding site in a cortical cell that was fused with adjacent cells forming the syncytium. Structurally, vascular syncytia varied in shape and expansion when induced by females or males. Those induced by females were larger and with more cytoplasmic contents compared to those induced by males. All syncytial cells were hypertrophic with dense cytoplasm and large nuclei. In some cases, nematode females penetrated into the cortex with the anterior elongated body portion where they induced a permanent feeding site and continued to remain in a semi-endoparasitic position, probably because of the nature of root tissues (woody host).

***Host-suitability test.*** The results presented in this paper as well as in the host-suitability test confirmed that *H.fici* has a narrow host range limited to *Ficus* species and for this reason it is not considered a major pest, as are other species of *Heterodera*.

**Figure 5.**
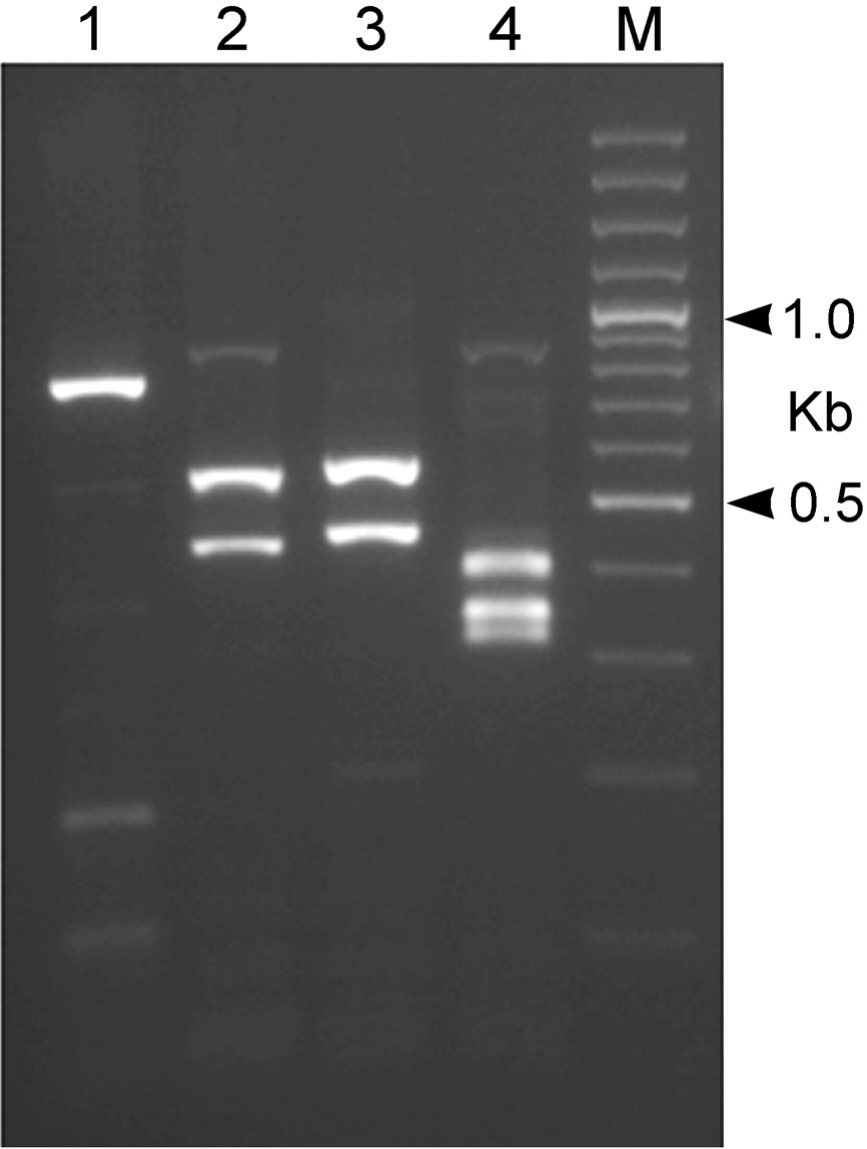
RFLP profiles of the ITS of *Heteroderafici* digested with four restriction enzymes. **M** 100 bp DNA ladder (Promega); **1** Alu I **2** Hae III **3** Rsa I **4** Pst I.

***Molecular characterization.*** The sequenced ITS, D2-D3 expansion domains of the 28S rRNA gene, and the 18S rRNA gene are 1035 bp, 780 bp, and 1627 bp long, respectively. BLAST search at NCBI revealed that the ITS, D2-D3 expansion domains of the 28S rRNA and the partial 18S rRNA sequences of *H.fici* from Italy, newly obtained in this study, matched well with the corresponding sequences of *H.fici* present in the database. The RFLP patterns of Italian *H.fici* were identical to those from Portugal, Greece, and another population from Potenza province, Basilicata, Italy (Madani et al. 2004) (Fig. 5). In particular, the ITS region of the Italian population of *H.fici* showed 99% similarity (1027/1029) with the corresponding region of *H.fici* deposited in GenBank, differing from 1 to 4 nucleotides; the next closest *Heterodera* species were *H.humuli* (coverage: 897/962), *H.vallicola* (coverage: 899/975), *H.ripae* (coverage: 894/972), and *H.litoralis* (coverage: 884/962) with 92–93% similarities. *Heterodera* species belonging to Schachtii group, instead, showed about 82% similarity with the Italian population of *H.fici*. The D2–D3 expansion domains of the Italian *H.fici*, obtained for the first time in this study, showed 95% similarity with the corresponding region of *H.latipons* Franklin, 1969 and *H.avenae* Wollenweber, 1924, while with *H.glycines* Ichinohe, 1952 and *H.schachtii* the similarity was 94%. The 18S rRNA gene of *H.fici* showed 99% similarity with the corresponding region of *H.avenae*, *H.hordecalis* Andersson, 1975, *H.schachtii*, and *H.glycines*, while its similarity with *H.elachista* Ohshima, 1974 was 98%.

**Figure 6.**
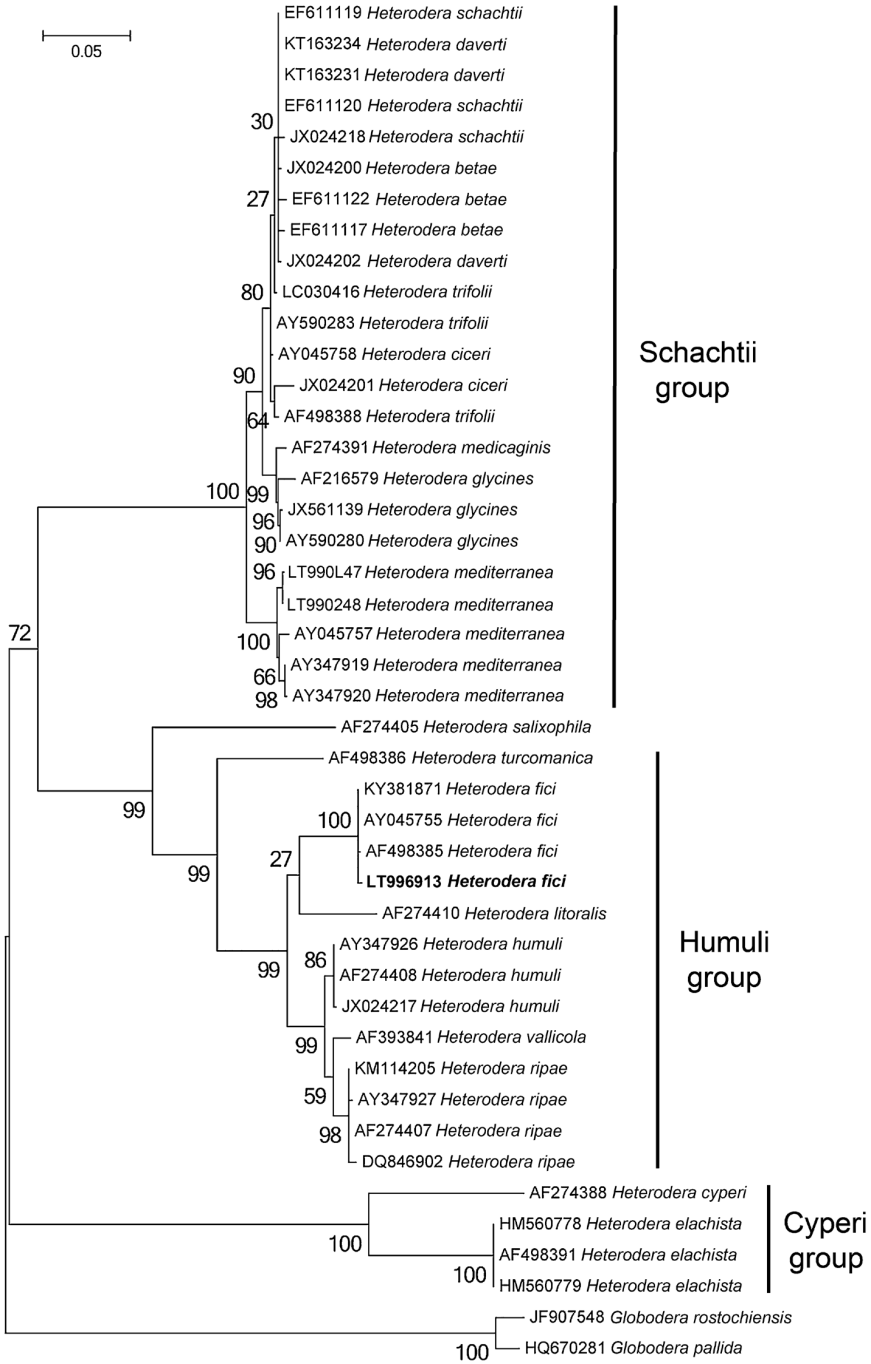
Phylogenetic trees of ITS containing region of *Heteroderafici* and the closest species. Sequences were analysed using the Maximum Likelihood method. Numbers at nodes indicate bootstrap values.

**Figure 7.**
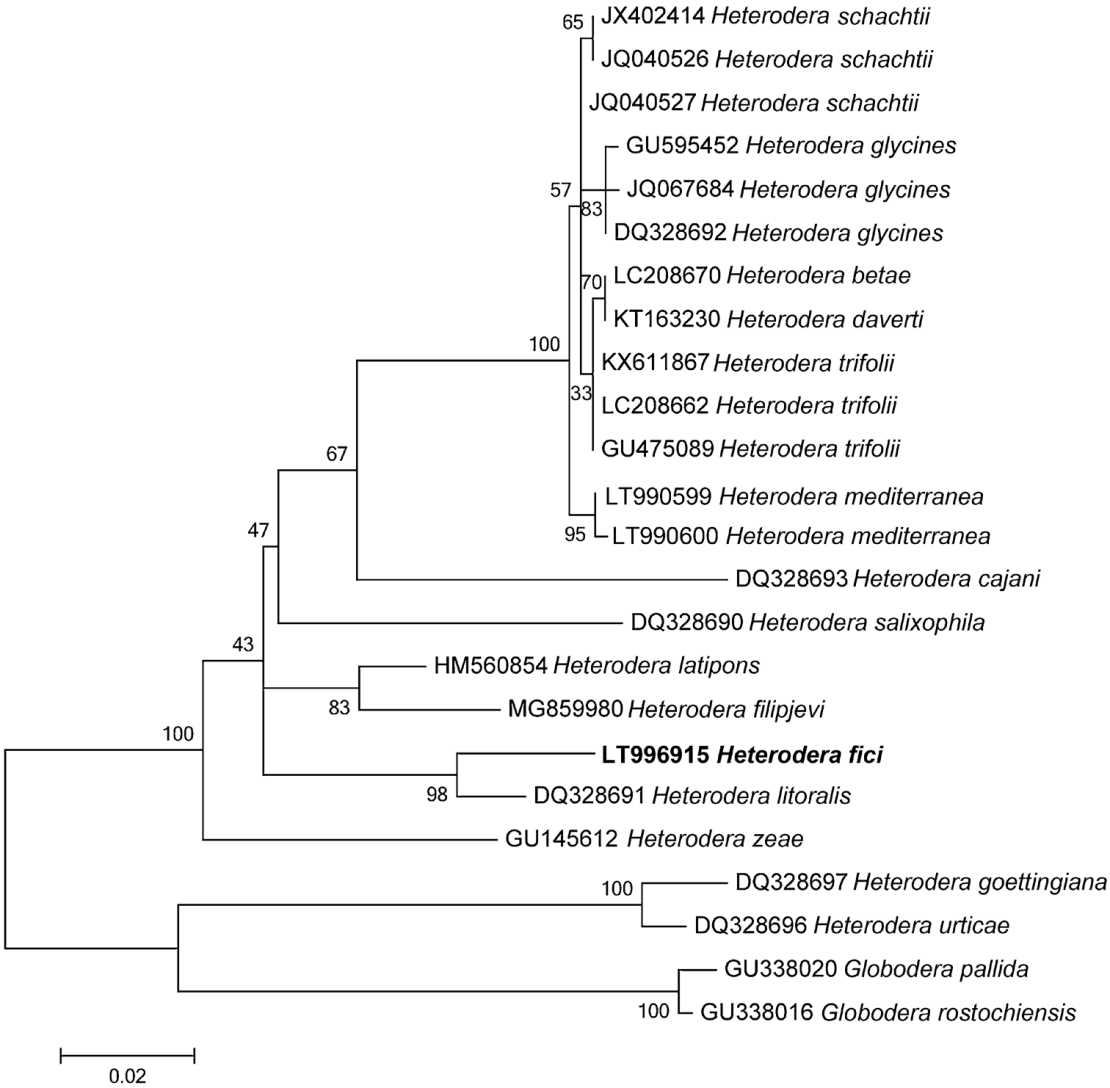
Phylogenetic trees of the D2–D3 expansion domains of the 28S rRNA gene of *Heteroderafici* and the closest species. Sequences were analysed using the Maximum Likelihood method. Numbers at nodes indicate bootstrap values.

**Figure 8.**
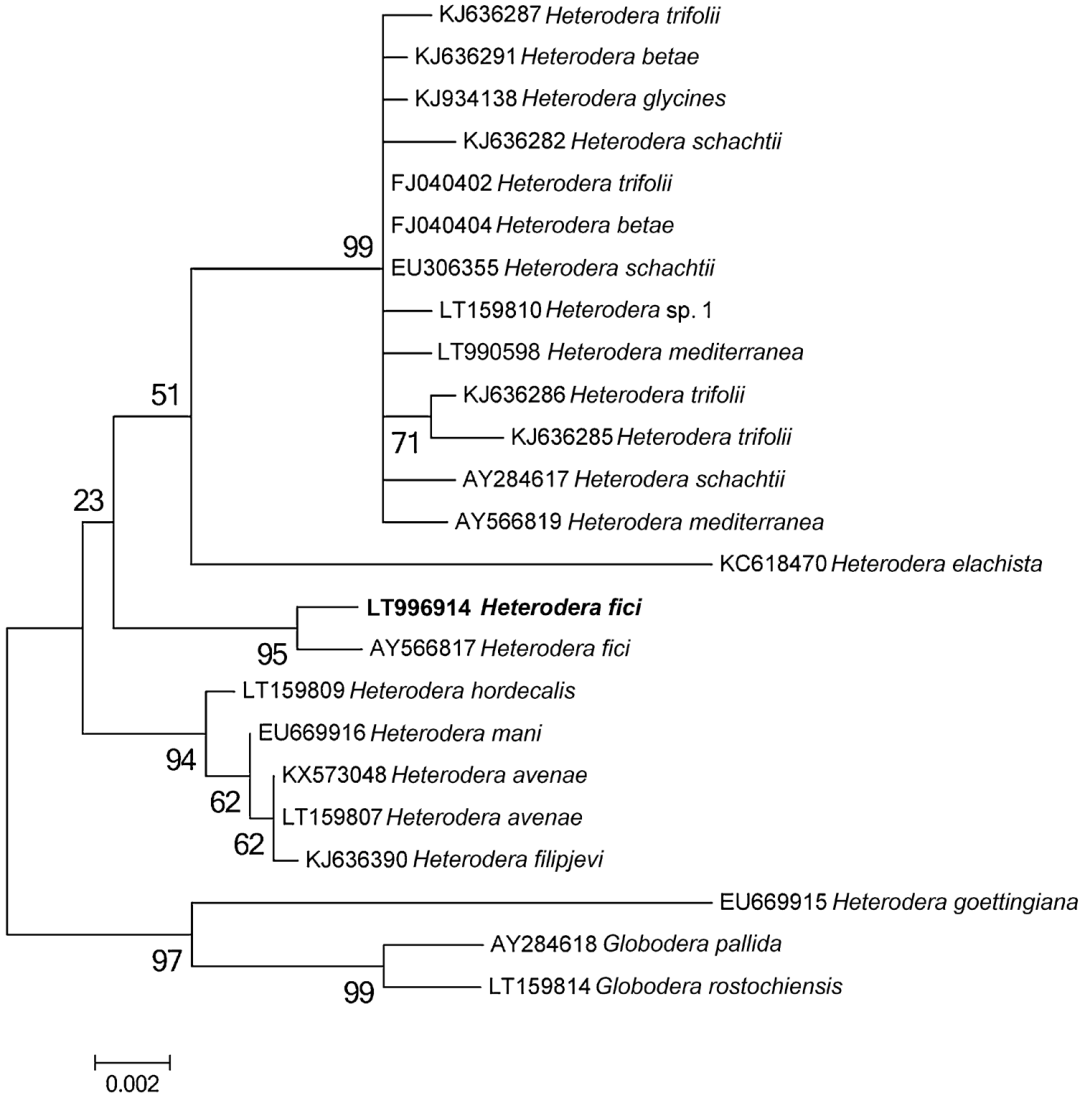
Phylogenetic trees of the 18S rRNA gene of *Heteroderafici* and the closest species. Sequences were analysed using the Maximum Likelihood method. Numbers at nodes indicate bootstrap values.

Phylogenetic trees using the ML method are given in Figures 6–8. ITS, D2-D3, and 18S trees confirmed the separation of the genus *Heterodera* into different groups (Subbotin et al. 2010, De Luca et al. 2013). In particular, the Italian population of *H.fici* grouped within the Humuli group in all phylogenetic trees, closely related to the Schachtii group.

## Discussion

Our study reports the occurrence of *H.fici* in commercial fig trees in southern Italy showing no symptoms of retarded growth or yellowing of leaves related to nematode presence. The morphology (Fig. 1) and the morphometrics (Table 1) of cysts, females, males, and second stage juveniles recovered from the roots and the crushed cysts of Italian *H.fici* agree with the original species description and subsequent redescription (Kirjanova 1954, Golden et al. 1988) except for minor intraspecific differences. According to the morphological and molecular data, the Italian population of *H.fici* shows some diagnostic characters similar to *Heterodera* species belonging to the Humuli group differing, however, from the members of this group (*H.humuli*, *H.ripae*, *H.vallicola*, and *H.litoralis*) by having ambifenestrate rather than bifenestrate cysts, longer vulval slit, the shape and measurements of J2 tails, and four prominent nipples on the male tail tip. Furthermore, SEM observations of cysts and males showed greater details of various structures compared to the light microscopy (Fig. 3). Infection of Italian *H.fici* on Mediterranean fruit trees revealed no symptoms when compared to infected fig roots. The results obtained in the survey, as well as in the host suitability test, confirmed that *H.fici* has a narrow host range limited to *Ficus* spp. and for this reason it is not considered a major pest as are other *Heterodera* spp. Furthermore, these findings also confirmed that *H.fici* is the only species of Humuli group attacking woody host plants; all the other members parasitize herbaceous hosts.

ITS-RFLP patterns of *H.fici* from South Italy showed no heterogeneity or differences to the published RFLP patterns (Madani et al. 2004). The phylogenetic relationships of *H.fici* obtained in this study reported in Figs 6–8 strongly support the monophyly of the genus *Heterodera* using *Globoderapallida* Stone, 1973 and *G.rostochiensis* (Wollenweber, 1923) Skarbilovich, 1959 as outgroups. Figure 6 shows the phylogenetic tree based on the ITS region confirming that *H.fici* grouped with all populations of *H.fici* and clustered with high bootstrap support (99%) in the Humuli group. Close relationships were revealed among the Humuli group, the species *H.salixophila* Kirjanova, 1969 and the Schachtii group, as already reported, confirming their coevolution with dicots (Subbotin et al. 2001). Furthermore, *H.fici* together with *H.vallicola* and *H.mediterranea* are the only *Heterodera* species known to attack woody plants belonging to different families, suggesting a host switch for these *Heterodera* species. The obtained phylograms are in agreement with the current morphological groupings of *Heterodera* species and coevolution with host plants.

In conclusion, our data confirm the occurrence of *H.fici* in two regions of southern Italy, Apulia and Basilicata, and on commercial fig orchards, approximately thirty years old, suggesting that this nematode despite its narrow host range is widespread all over the world and that it deserves attention. Maqbool et al. (1987) reported yellowing of fig trees in Pakistan but they were infested with both *Meloidogynejavanica* (Treub, 1885) Chitwood, 1949 and *H.fici* and, therefore, it was not possible to partition the observed damage between the two nematodes. However, during our survey *H.fici* was detected in rather large population densities both in soil and roots and appeared widespread (25% of the orchard infested). Therefore, although the impact of *H.fici* on fig yield still remains to be assessed, we believe that *H.fici* deserves attention to avoid its spread and subsequent yield loss.

## Supplementary Material

XML Treatment for Heterodera fici
